# Determinants of Visual Impairment Among Chinese Middle-Aged and Older Adults: Risk Prediction Model Using Machine Learning Algorithms

**DOI:** 10.2196/59810

**Published:** 2024-10-09

**Authors:** Lijun Mao, Zhen Yu, Luotao Lin, Manoj Sharma, Hualing Song, Hailei Zhao, Xianglong Xu

**Affiliations:** 1 School of Public Health, Shanghai University of Traditional Chinese Medicine, 1200 Cai Lun Road, Shanghai, 201203, China, 86 18721538966, 86 021-51322421; 2 Monash e-Research Centre, Faculty of Engineering, Airdoc Research, Nvidia AI Technology Research Centre, Monash University, Melbourne, Australia; 3 Nutrition and Dietetics Program, Department of Individual, Family, and Community Education, University of New Mexico, Albuquerque, NM, United States; 4 Department of Social and Behavioral Health, School of Public Health, University of Nevada, Las Vegas, NV, United States; 5 Department of Internal Medicine, Kirk Kerkorian School of Medicine, University of Nevada, Las Vegas, NV, United States; 6 School of Translational Medicine, Faculty of Medicine, Nursing and Health Sciences, Monash University, Melbourne, Victoria, Australia; 7 Artificial Intelligence and Modelling in Epidemiology Program, Melbourne Sexual Health Centre, Alfred Health, Carlton, Victoria, Australia

**Keywords:** visual impairment, China, middle-aged and elderly adults, machine learning, prediction model

## Abstract

**Background:**

Visual impairment (VI) is a prevalent global health issue, affecting over 2.2 billion people worldwide, with nearly half of the Chinese population aged 60 years and older being affected. Early detection of high-risk VI is essential for preventing irreversible vision loss among Chinese middle-aged and older adults. While machine learning (ML) algorithms exhibit significant predictive advantages, their application in predicting VI risk among the general middle-aged and older adult population in China remains limited.

**Objective:**

This study aimed to predict VI and identify its determinants using ML algorithms.

**Methods:**

We used 19,047 participants from 4 waves of the China Health and Retirement Longitudinal Study (CHARLS) that were conducted between 2011 and 2018. To envisage the prevalence of VI, we generated a geographical distribution map. Additionally, we constructed a model using indicators of a self-reported questionnaire, a physical examination, and blood biomarkers as predictors. Multiple ML algorithms, including gradient boosting machine, distributed random forest, the generalized linear model, deep learning, and stacked ensemble, were used for prediction. We plotted receiver operating characteristic and calibration curves to assess the predictive performance. Variable importance analysis was used to identify key predictors.

**Results:**

Among all participants, 33.9% (6449/19,047) had VI. Qinghai, Chongqing, Anhui, and Sichuan showed the highest VI rates, while Beijing and Xinjiang had the lowest. The generalized linear model, gradient boosting machine, and stacked ensemble achieved acceptable area under curve values of 0.706, 0.710, and 0.715, respectively, with the stacked ensemble performing best. Key predictors included hearing impairment, self-expectation of health status, pain, age, hand grip strength, depression, night sleep duration, high-density lipoprotein cholesterol, and arthritis or rheumatism.

**Conclusions:**

Nearly one-third of middle-aged and older adults in China had VI. The prevalence of VI shows regional variations, but there are no distinct east-west or north-south distribution differences. ML algorithms demonstrate accurate predictive capabilities for VI. The combination of prediction models and variable importance analysis provides valuable insights for the early identification and intervention of VI among Chinese middle-aged and older adults.

## Introduction

Visual impairment (VI) represents a significant global public health challenge. Over the period from 1990 to 2019, the burden index of VI has escalated for individuals aged 50‐74 years and individuals aged 75 years and older, shifting from the 20th and 16th positions to the 19th and 15th positions, respectively [1]. The global increase in VI prevalence is primarily attributed to cataracts and uncorrected refractive errors, accounting for 55% of blindness cases and 77% of VI cases among adults aged 50 years and older in 2015 [2]. This trend is further exacerbated by population growth and aging [2]. According to the National Bureau of Statistics of China’s January 2022 report, adults aged 60 years and older accounted for 18.9% of the total population by the end of 2021.

Meanwhile, adults aged 65 years and older exceeded 200 million, representing 14.2% of the total population [3]. It is projected that during the “14th Five-Year Plan” period, the total number of adults aged 60 years and older will surpass 300 million, accounting for over 20% of the population, indicating a transition into the moderate aging phase. By around 2035, China is anticipated to enter the severe aging phase [3]. Older adults with VI are at a heightened risk of falls [4], potentially leading to fractures and severe outcomes such as cerebral hemorrhage. Furthermore, VI can hinder social engagement among older adults, possibly giving rise to more profound mental health issues, including depression and anxiety [5]. As the population ages, the prevalence of VI is expected to increase dramatically. However, half of all VI cases are estimated to be preventable or treatable [6]. Hence, bolstering screening efforts and enhancing risk prediction for VI is paramount in stemming the tide of this growing concern.

In response to the crises posed by VI and to improve public visual health, the China National Health Commission has released the 14th Five-Year National Eye Health Plan (2021‐2025). The plan focuses on enhancing eye health information platforms and promoting the harmonious integration of big data, artificial intelligence, and ophthalmology services to advance the early detection of eye diseases [7]. Through the development of machine learning (ML) prediction models for VI, the precise determination of VI risk and identification of influencing risk factors can be achieved. ML could offer new insights for early detection and timely intervention of retinopathy, as well as for the integrated management of ocular health in older adults, ultimately enhancing the overall eye health status of the population.

Artificial intelligence has experienced swift progress in recent years, resulting in extensive use of diverse ML algorithms in clinical research [8,9]. Compared with traditional statistical methods, ML algorithms can handle more complex nonlinear relationships, interactions, and multiple covariances, significantly improving the predictive ability of artificial intelligence models [10,11].

Despite the advantages of ML algorithms, there is an absence of the use of ML algorithms to predict the risk of VI in the general middle-aged and older adult population in China. Previous studies on predicting VI have focused on various topics, including examining trends in the incidence of VI among populations [12,13]; assessing the risk of VI in specific groups, such as those with congenital cytomegalovirus infection [14-16]; and further predicting the risk of developing particular types of VI [17]. However, these studies have not focused on predicting the individual risk of VI among the general population. In current research on predicting individual risk of VI among the general population, three studies [18-20] based on traditional statistical methods and two studies [21,22] based on ML algorithms have achieved good predictive performance. Among these, two studies [20,21] lacked Chinese populations, with the studied populations being mainly from the United States and Singapore; one focused on Chinese children [22]; one was a single-center study [18]; and one [19] had a small sample size of 133 participants. To date, no research has yet been conducted on using ML algorithms to predict VI in Chinese middle-aged and older adults. Therefore, our objective was to develop an individual risk prediction model for VI, which could be used to assess the risk of VI among China’s general middle-aged and older population. Additionally, we aimed to identify key predictors of VI. Our findings could be used to provide personalized intervention guidance for health care professionals, aiming to reduce and delay the onset of retinal diseases among middle-aged and older adults.

## Methods

### Analytic Sample

The data used in our study originate from the China Health and Retirement Longitudinal Study (CHARLS), a longitudinal survey that represents a nationally diverse cohort of Chinese adults aged 45 years and older. This survey strives to establish a comprehensive public database documenting Chinese adults’ social, economic, and health statuses, thereby bolstering scientific investigations conducted by the National Development Institute of Peking University. The CHARLS project executed a nationwide baseline survey between 2011 and 2012, with subsequent follow-up visits occurring biennially [23]. The CHARLS baseline survey encompassed 450 villages and neighborhoods spread across 150 counties in China. The sampling process encompassed multiple levels, including counties, villages, households, and individuals, culminating in interviews with 10,257 households and broadly reflecting the general Chinese middle-aged and older adult populace. We used 25,538 observations from 4 waves of surveys between 2011 and 2018. Excluding ages younger than 45 years (n=4079) and missing information on self-reported vision conditions (n=2457), 19,047 participants were included in this study. More details of the sampling process are shown in Figure S1A in Multimedia Appendix 1.

### Predictors of VI

Drawing on existing literature and expert insights, 42 predictors were used for ML algorithm training. The predictors were categorized as follows: self-reported questionnaire, physical examination, and blood biomarkers. The self-reported questionnaire included (1) demographic factors (gender, age, and region), [24] (2) lifestyle (night sleep duration, smoking, and drinking), (3) health status factors (pain, weight change, health status during childhood, and self-expectations of health status), [25] (4) disease factors (depression, hearing impairment, hypertension, dyslipidemia, diabetes, liver disease, heart disease, stroke, kidney disease, stomach or other digestive disease, memory-related disease, arthritis or rheumatism, menopause, and prostatic diseases), (5) living environment factors (house structure, heating energy, cooking energy, and room temperature), and (6) socioeconomic factors (standard of living and education level). Measurement parameters included (1) physical examination data ([26] hand grip strength, waist, and BMI) and (2) blood biomarker data (white blood cell, platelets, glycated hemoglobin, glucose, total cholesterol, triglycerides, high-density lipoprotein cholesterol, and low-density lipoprotein cholesterol). The characteristics of the predictor distribution in the study are displayed in Table S1 in Multimedia Appendix 1.

### Measurement of VI

Vision encompasses both far and near eyesight. VI in our study was assessed using the following questions from the CHARLS questionnaire: (1) “How well do you see things in the distance? For example, can you recognize a friend across the road (even with glasses on)? Is it excellent, very good, good, fair, or bad?” and (2) “How well do you see things close up? For example, can you read a newspaper with your glasses on? Is it excellent, very good, good, fair, or bad?” Respondents who answered “not good” to any of the questions were categorized as having a VI, while those who answered “excellent” to “fair” were considered to have no VI [27].

### Statistical Analysis

We used R (version 4.3.1), developed by the R Core Team of the R Foundation for Statistical Computing, for statistical analysis and model development. The summary of continuous variables involved the use of the median and IQR (25th and 75th percentiles), and categorical variables were summarized by providing the count (n) and proportion (%) for each category. We used the R H2O package to construct various ML predictive models for a dichotomous outcome of VI [28]. H2O supports a wide range of ML models, including deep learning (DL), gradient boosting machine (GBM), distributed random forest (DRF), and more. In our study, we chose the generalized linear model (GLM) as our benchmark model, representing logistic regression. The study process is shown in Figure S1B in Multimedia Appendix 1. As per the No Free Lunch Theorem [29], no algorithm can outperform a linear enumeration of the search space or a purely random search algorithm. Thus, we split the dataset into training (n=14,286) and testing (n=4761) datasets at a 75:25 ratio. The training dataset was used to develop various models, including a GLM with regularization to prevent overfitting and enhance the model’s predictive accuracy. GBM uses decision trees as weak learners and boosts their predictions iteratively [30]. DRF incorporates both DRF and extremely randomized tree approaches to ensure diversity and robustness in the ensemble [31]. The DL model consists of a fully connected multilayer artificial neural network trained with backpropagation to capture complex nonlinear relationships [32,33]. A stacked ensemble combines the predictions of these individual models as input features for the ensemble’s meta-learner. The meta-learner then outputs a final prediction based on the learned weights of each base model’s contribution, enhancing overall prediction performance [34]. The random forest algorithm was used to impute missing values, while the continuous variables were normalized. In this study, the ratio of positive to negative outcomes in the target variable was 1:2, indicating an imbalanced dataset. To address data imbalance, random oversampling of the minority class was initially used [35]. Furthermore, to mitigate overfitting and enhance model generalization [36], external 5-fold cross-validation was implemented. However, the model’s performance did not show improvement compared with the no-resampling, blending mode. Therefore, we trained the stacked combinations using the no-resampling and blending mode, plotted the receiver operating characteristic (ROC) curves, and constructed the confusion matrix. We used the area under the curve (AUC) to evaluate the best model, with an acceptable AUC of 0.7‐0.8, a good AUC of 0.8‐0.9, and an excellent AUC of >0.9 [37]. We calibrated the probabilities predicted by the models to the actual occurrence level in the testing dataset using a logistic function and calculated the Brier score to assess the reliability of the prediction of VI [38]. The Brier score takes values from 0 to 1, and at a predicted probability of 50%, the Brier score is 0.25 [39]. A model score between 0 and 0.25 indicates a correct prediction, and a score closer to 0 indicates better model effectiveness. Additionally, we used models with acceptable AUCs for variable importance analysis. This enabled us to quantitatively assess the contribution of each feature towards model predictions, thereby allowing us to evaluate and compare the significance of various features.

### Ethical Considerations

The Peking University Institutional Review Board (IRB) granted ethical approval for all waves of the CHARLS. The IRB approval number for the self-reported questionnaire (including physical examination measurements) was IRB00001052-11015; the IRB approval number for the biomarker collection was IRB00001052-11014.

## Results

### Geographical Distribution of VI

**Figure 1.**
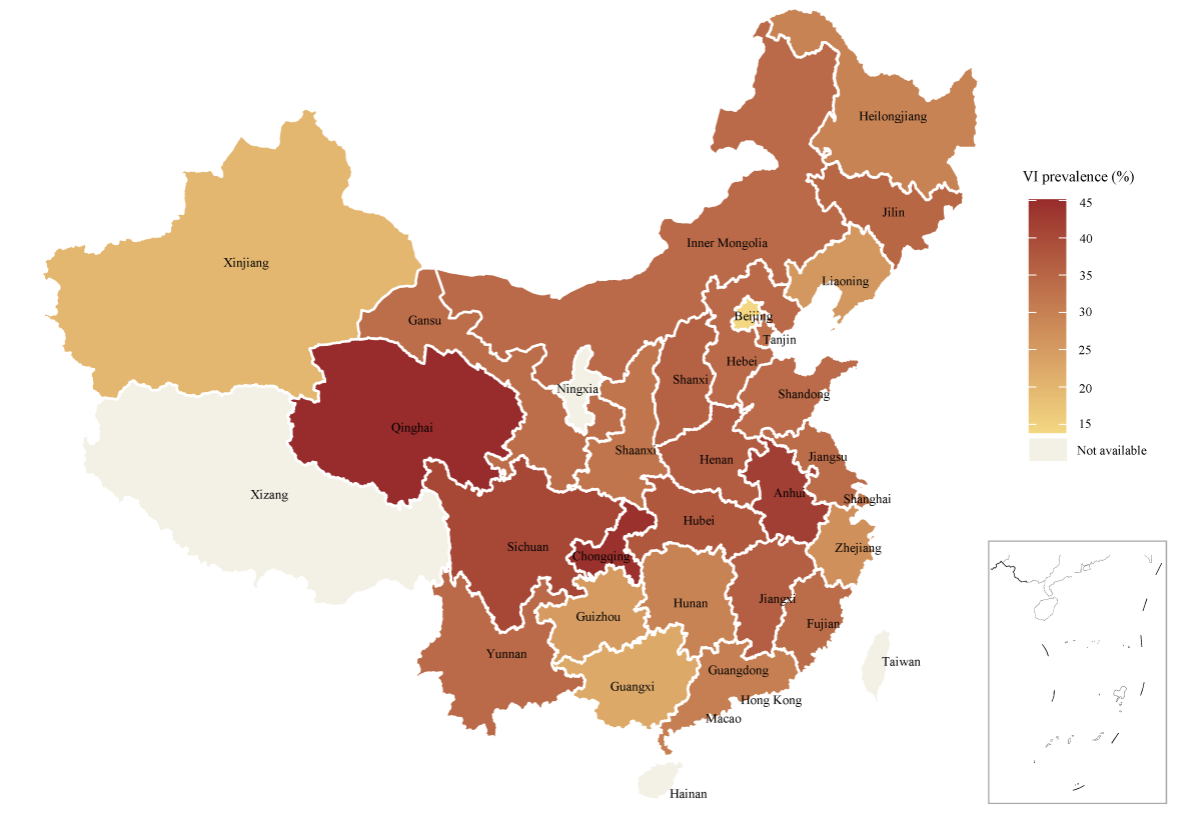
The prevalence of VI by province in China from the China Health and Retirement Longitudinal Study (2011‐2018) 4 waves. VI: visual impairment.

Figure 1 presents the prevalence of VI by province in China, based on data from the 4 waves of the CHARLS conducted between 2011 and 2018. Qinghai, Chongqing, Anhui, and Sichuan provinces reported a high prevalence of VI, with rates exceeding 40% (69/153, 45.1%; 117/265, 44.2%; 390/930, 41.9%; and 654/1619, 40.4%, respectively). In contrast, Xinjiang and Beijing had a low prevalence of VI, with rates below 20% (23/116, 19.8% and 14/101, 13.8%, respectively). The remaining provinces, municipalities, and autonomous regions exhibited a moderate prevalence of VI, ranging from 20% to 40%. Additional information regarding the prevalence of VI in each province is presented in Table S2 in Multimedia Appendix 1.

### Characteristics of the Study Participants

A total of 33.9% (6449/19,047) of participants reported VI, divided between the training dataset (n=4837) and the testing dataset (n=1612). Among the cases of VI, 58.8% (3795/6449) were female, and 41.2% (2654/6449) were male. The age group with the highest prevalence of VI was 55‐65 years, accounting for 39.1% (2520/6449), followed by those aged 65 years or older at 31.4% (2025/6449) and 45‐55 years at 29.5% (1904/6449). The selected characteristics of the study participants are shown in Table 1. The full characteristics of the predictor distribution in the study are shown in Table S1 in Multimedia Appendix 1.

**Table 1.** Selected characteristics of study participants among Chinese adults older than 45 years as drawn from the China Health and Retirement Longitudinal Study (2011‐2018; N=19,047).

Characteristic		Overall (N=19,047), n (%)	Non-VI^a^ (n=12,598), n (%)	VI (n=6449), n (%)	*P* value^b^
**Sex**					<.001
	Female	9927 (52.1)	6132 (48.7)	3795 (58.8)	
	Male	9120 (47.9)	6466 (51.3)	2654 (41.2)	
**Age (years)**					<.001
	45‐55	7279 (38.2)	5375 (42.7)	1904 (29.5)	
	55‐65	6794 (35.7)	4274 (33.9)	2520 (39.1)	
	≥65	4974 (26.1)	2949 (23.4)	2025 (31.4)	
**Region**					<.001
	East	6995 (36.7)	4486 (35.6)	2509 (38.9)	
	Central	6315 (33.2)	4355 (34.6)	1960 (30.4)	
	West	5737 (30.1)	3757 (29.8)	1980 (30.7)	
**Education level**					<.001
	Less than elementary school	8368 (43.9)	4869 (38.6)	3499 (54.3)	
	Elementary school	4113 (21.6)	2805 (22.3)	1308 (20.3)	
	Middle school	3971 (20.8)	2909 (23.1)	1062 (16.5)	
	High school or above	2595 (13.6)	2015 (16.0)	580 (9.0)	
**Standard of living**					<.001
	Poor	2260 (11.9)	1182 (9.4)	1078 (16.7)	
	Relatively poor	5824 (30.6)	3768 (29.9)	2056 (31.9)	
	Average	10,420 (54.7)	7257 (57.6)	3163 (49.0)	
	Relatively high	507 (2.7)	365 (2.9)	142 (2.2)	
	Very high	36 (0.2)	26 (0.2)	10 (0.2)	

a VI: visual impairment.

b Pearson *χ*2 test.

### VI Prediction

**Figure 2.**
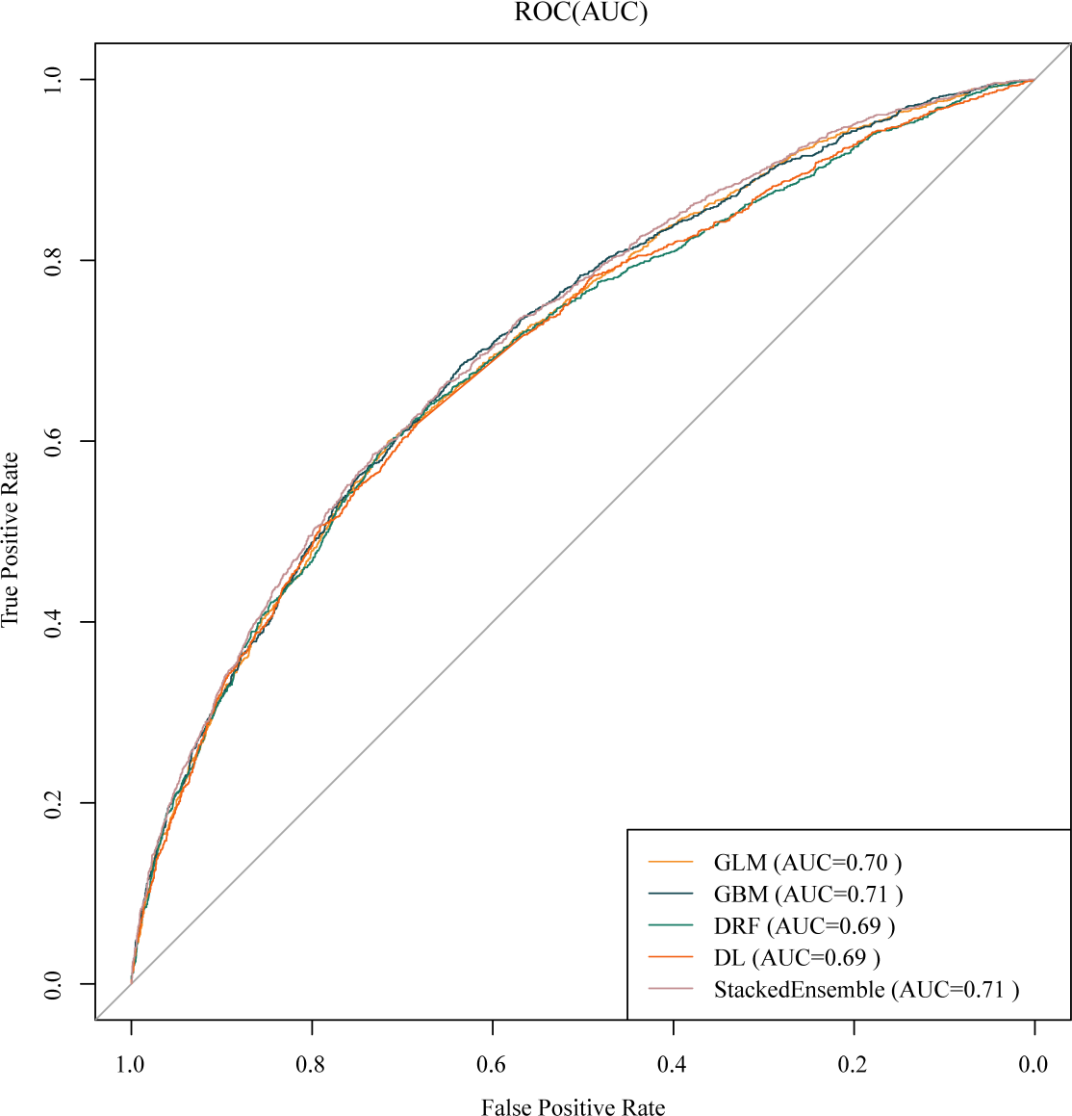
Receiver operating characteristic curves of all visual impairment prediction models on the testing dataset. AUC: area under the curve; DL: deep learning; DRF: distributed random forest; GBM: gradient boosting machine; GLM: generalized linear model; ROC: receiver operating characteristic; StackedEnsemble: GBM-XGBoost-GLM-DL-DRF.

We applied the trained models to the testing dataset. The distribution of predictor variables between the testing and training datasets is detailed in Table S3 in Multimedia Appendix 1. The results indicate that the ensemble model demonstrates superior predictive performance compared with individual ML models. Three algorithms, namely the GLM, GBM, and stacked ensemble model (GBM-XGBoost-GLM-DL-DRF), achieved acceptable AUC values 0.706, 0.710, and 0.715, respectively. The ensemble model exhibited the best performance. However, the DRF and DL models did not meet the acceptable AUC threshold of 0.70, achieving an AUC of 0.698. Detailed evaluation metrics for all models on the testing dataset are provided in Table S4 in Multimedia Appendix 1. Figure 2 depicts the ROC curves for all the models. The hyperparameters used in model training are summarized in Table S5 in Multimedia Appendix 1. ROC curves of all VI prediction models on the training dataset are shown in Figure S2 in Multimedia Appendix 1.

### Assessing the Efficacy of ML Models for VI Prediction

**Figure 3.**
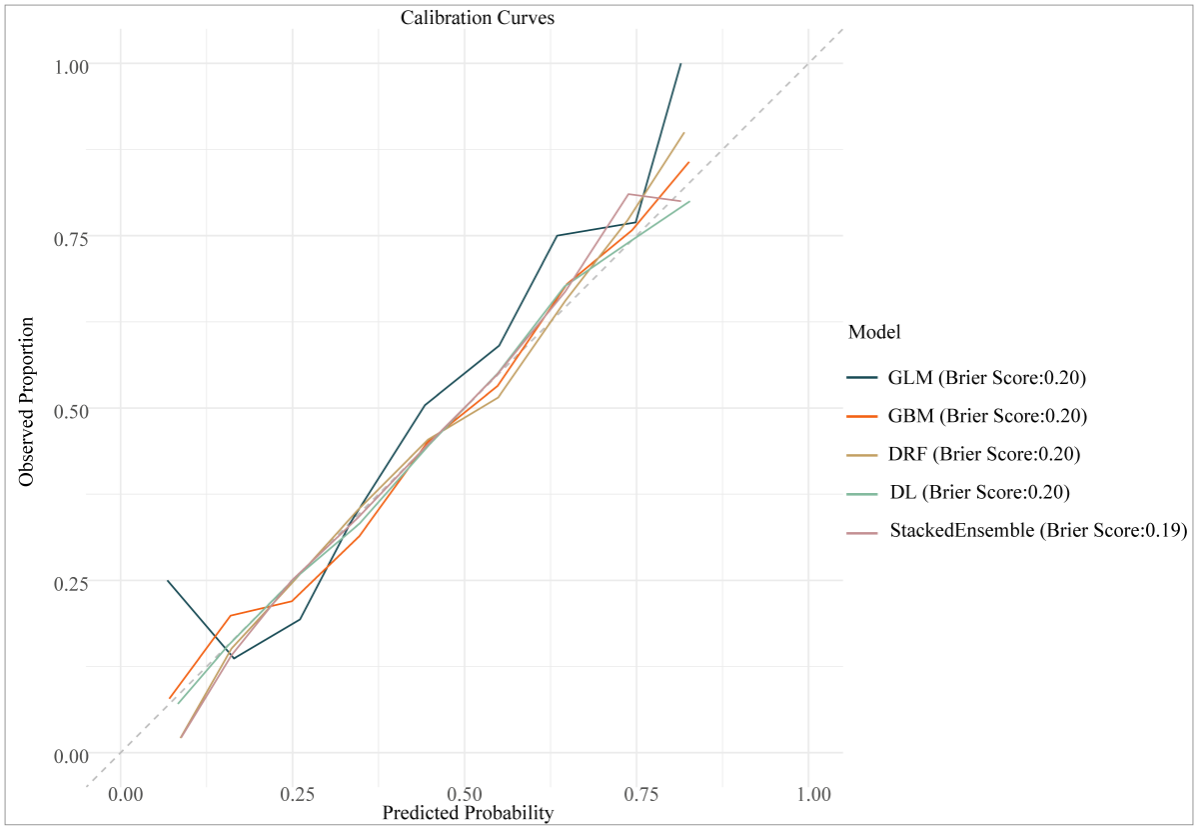
The calibration curves of all visual impairment prediction models on the testing dataset. DL: deep learning; DRF: distributed random forest; GBM: gradient boosting machine; GLM: generalized linear model; StackedEnsemble: GBM-XGBoost-GLM-DL-DRF.

Figure 3 presents the calibration curves of all the models on the testing dataset. These curves illustrate the agreement between each model’s predicted probabilities and VI’s observed probabilities in the testing data. The results indicated that all models accurately predicted VI, as evidenced by their Brier scores being less than 0.25.

### Determinants of VI

Table 2 presents the importance of predictors for VI in models with acceptable AUCs. The top 10 important predictors for VI were identified by the GLM, including hearing impairment, pain, depression, hand grip strength, standard of living, education level, age, self-expectation of health status, night sleep duration, and arthritis or rheumatism. For GBM, the top predictors for VI were hearing impairment, self-expectation of health status, pain, age, hand grip strength, depression, night sleep duration, hemoglobin, high-density lipoprotein cholesterol, and arthritis or rheumatism. Notably, hearing impairment, self-expectation of health status, pain, age, hand grip strength, depression, night sleep duration, and arthritis or rheumatism have emerged as common predictors for these models.

**Table 2.** Variable importance analysis was performed by the generalized linear model (GLM) and gradient boosting machine (GBM), sorted in descending order.

Rank	GLM	GBM
1	Hearing impairment	Hearing impairment
2	Pain	Self-expectations of health status
3	Depression	Pain
4	Hand grip strength	Age
5	Standard of living	Hand grip strength
6	Education level	Depression
7	Age	Night sleep duration
8	Self-expectations of health status	Hemoglobin
9	Night sleep duration	High-density lipoprotein cholesterol
10	Arthritis or rheumatism	Arthritis or rheumatism

## Discussion

### Principal Results

To our knowledge, this study is the first attempt to predict the risk of VI among middle-aged and older adults in China using ML algorithms. The findings indicated that ML algorithms could accurately identify individuals at risk of VI among this demographic. Our results also showed that ensemble algorithms proved superior to individual ML models. Furthermore, we calculated the prevalence of VI and presented it through regional visualization. The results indicated the existence of regional disparities in the prevalence of VI among Chinese middle-aged and older adults, with varying rates across provinces. However, it is worth noting that we did not find any significant north-south or east-west directional differences in prevalence. Based on these results, gaining a more intuitive understanding of the regional distribution of VI is possible. Additionally, our prediction model can be leveraged to develop a risk assessment tool for early detection of VI. Predictors’ importance could help guide personalized early interventions for middle-aged and older individuals at risk of VI.

### Comparison With Prior Work

Our study showed that GBM outperformed logistic regression in predicting VI among middle-aged and older Chinese adults. Previous ML studies on VI prediction have predominantly relied on single algorithms without comparing the predictive performance across multiple algorithms. In contrast, our work used various ML algorithms for VI prediction and assessed their comparative effectiveness. Furthermore, our findings highlighted the superior performance of ensemble algorithms over individual learning models despite an accuracy of 0.625. This is due to the imbalanced nature of our dataset, where accuracy may not fully reflect the model’s ability to predict VI. We chose AUC as the primary metric and found that the ensemble model had the highest AUC, indicating its overall superior ability to rank positive and negative samples.

The calibration curves showed satisfactory calibration but moderate discrimination, indicating the models’ ability to assess the overall risk for VI in the population effectively. This finding has significant implications for designing resource allocation strategies in clinical and public health settings. While the models’ discriminatory power was not exceptional, they could be used as supplementary tools in broader clinical or public health assessment frameworks, providing valuable insights for early screening of VI risk. Nevertheless, we acknowledged and considered the limitations of these models when applying them in practice. Consequently, our research contributed significantly to future investigations of ML algorithms for VI prediction by providing a comprehensive evaluation of multiple algorithms and emphasizing the advantages of ensemble methods. Additionally, our work offers technical guidance for the primary prevention of VI by identifying the most effective predictive models.

Our study has the following strengths compared with previous ML models for VI prediction. First, our model is more adept at identifying potentially modifiable risk factors. Unlike prior studies that primarily relied on image or video data as predictors [21,22], our approach incorporates easily accessible everyday information, such as lifestyle factors like night sleep duration. Second, our predictive model holds greater representativeness and general applicability for the middle-aged and older adult Chinese population. Previous domestic studies on VI prediction tended to focus on specific disease groups (stroke patients) or particular types of VI (anterior retinal VI) [14,15]. Our study, based on a national sample, includes individuals from the middle-aged and older adult demographics, and our results pertain to general types of VI.

We found that the important predictors of VI included hearing impairment, self-expectation of health status, pain, age, hand grip strength, depression, night sleep duration, high-density lipoprotein cholesterol, and arthritis or rheumatism. This finding aligns with those documented in prior research [24,25,40-42]. Significantly, our study newly highlighted the crucial role of hearing impairment, self-expectation of health status, and pain in predicting VI. Although hearing impairment does not directly affect vision, its underlying causes may be associated with ocular or neurological disorders. For instance, neurofibromatosis occurring near the inner ear and optic nerve can potentially lead to concurrent hearing and VI [43]. The eyes and ears share a common neuroectodermal origin and exhibit similar genetic networks [44]. When pathogenic mutations occur in these shared genes, they can concurrently affect the functions of both the eyes and ears, leading to dual sensory loss. For instance, defects in the development of inner ear hair cells and photoreceptor cells may underlie the pathogenesis of Usher syndrome, the most prevalent syndromic form of retinitis pigmentosa [45]. In addition, self-expectation of health status reveals one’s attitudes towards personal health. High expectations often lead to proactive health behaviors, such as regular ophthalmologic check-ups for vision issues. In contrast, lower expectations, possibly due to comorbid chronic conditions [25], may cause psychological stress affecting vision [46]. For example, depression is more prevalent in patients with VI [47]. Additionally, pain serves as a vital physiological indicator, revealing certain discomforts or underlying ailments in the body. Prolonged pain can keep an individual in a constant state of stress, disrupting the normal functions of the immune and endocrine systems, thereby indirectly impacting vision. Our findings enhance comprehension of the mechanisms underlying the development of VI, enabling early identification of high-risk groups and the implementation of targeted interventions for these individuals. Moreover, our findings provide a valuable reference for selecting variables in constructing VI prediction models. Nevertheless, to ensure the accuracy and credibility of our findings, further studies are required to validate these associations.

### Limitations

This study has several limitations that should be acknowledged. First, the assessment of VI relied on self-reported data, which may be subject to recall bias or subjectivity in responses. The qualitative nature of the VI assessment responses lacks the numerical precision of a quantitative evaluation, potentially affecting the accuracy of the outcome variable. Second, while environmental and lifestyle factors were considered, VI is also influenced by genetic factors, which were not included in the model due to the absence of such information in the database. Incorporating genetic data could potentially enhance the model’s predictive performance. Finally, the data were sourced from the CHARLS, which only represents the Chinese population aged 45 years and older. Consequently, the model’s generalizability to other age groups or populations outside of China remains uncertain. Further validation studies are necessary to evaluate the model’s effectiveness in diverse populations across different countries and age ranges.

### Conclusions

The prevalence of VI was notably high among middle-aged and older Chinese adults, displaying regional disparities but no significant variances between north-south or east-west regions. Our study is the first to use ML algorithms in predicting VI among China’s general middle-aged and older population. The findings demonstrate that ML algorithms can accurately predict VI among this demographic. Ensemble algorithms outperform individual learning models in predicting VI. Variable importance analysis highlighted the importance of considering factors such as hearing impairment and individuals’ self-expectation of health status when predicting VI risk. By incorporating these predictors, our study facilitates the early identification of individuals at high risk for VI, enabling timely interventions and preventive measures to mitigate the development and progression of VI.

## Supplementary material

10.2196/59810 Multimedia Appendix 1 Supplementary tables and figures containing further data on participant characteristics, visual impairment prevalence, predictive factors, model performance, hyperparameters, the sampling and study processes, and receiver operating characteristic curves of prediction models on the training dataset.

## Abbreviations

AUC: area under the curve
CHARLS: China Health and Retirement Longitudinal Study
DL: deep learning
DRF: distributed random forest
GBM: gradient boosting machine
GLM: generalized linear model
IRB: Institutional Review Board
ML: machine learning
ROC: receiver operating characteristic
VI: visual impairment
