# Supplementary material for: Determinants of Visual Impairment Among Chinese Middle-Aged and Older Adults: Risk Prediction Model Using Machine Learning Algorithms
Source: JMIR Aging. 2024 Oct 9;7:e59810. doi: 10.2196/59810 (PMC11481821; doi:10.2196/59810)
Supplement: Multimedia Appendix 1 [file aging-v7-e59810-s001.docx]

Appendix List

**Table S1.** Characteristics of study participants.

**Table S2.** The prevalence of VI by province in China from CHARLS (2011-2018) four waves.

**Table S3.** Analysis of differences in predictive factors between testing and training datasets.

**Table S4.** Comparison of machine learning model performance (testing dataset).

**Table S5.** The hyperparameters used in model training.

**Figure S1.** Flowchart of this study.

**Figure S2.** Receiver operating characteristic curves of all VI prediction models on the training dataset.

**Table S1.** Characteristics of study participants.

| Characteristic | Overall, N = 19,047^a^ | Non-VI, N = 12,598^a^ | VI, N = 6,449^a^ | *p*-value^b^ |
| --- | --- | --- | --- | --- |
| **Gender** |  |  |  | <.001 |
| Female | 9,927(52.1%) | 6,132(48.7%) | 3,795(58.8%) |  |
| Male | 9,120(47.9%) | 6,466(51.3%) | 2,654(41.2%) |  |
| **Age** |  |  |  | <.001 |
| 45-55 | 7,279(38.2%) | 5,375(42.7%) | 1,904(29.5%) |  |
| 55-65 | 6,794(35.7%) | 4,274(33.9%) | 2,520(39.1%) |  |
| ≥65 | 4,974(26.1%) | 2,949(23.4%) | 2,025(31.4%) |  |
| **Region** |  |  |  | <.001 |
| East | 6,995(36.7%) | 4,486(35.6%) | 2,509(38.9%) |  |
| Central | 6,315(33.2%) | 4,355(34.6%) | 1,960(30.4%) |  |
| West | 5,737(30.1%) | 3,757(29.8%) | 1,980(30.7%) |  |
| **Education level** |  |  |  | <.001 |
| Less than elementary school | 8,368(43.9%) | 4,869(38.6%) | 3,499(54.3%) |  |
| Elementary school | 4,113(21.6%) | 2,805(22.3%) | 1,308(20.3%) |  |
| Middle school | 3,971(20.8%) | 2,909(23.1%) | 1,062(16.5%) |  |
| High school or above | 2,595(13.6%) | 2,015(16.0%) | 580(9.0%) |  |
| **Standard of living** |  |  |  | <.001 |
| Poor | 2,260(11.9%) | 1,182(9.4%) | 1,078(16.7%) |  |
| Relatively poor | 5,824(30.6%) | 3,768(29.9%) | 2,056(31.9%) |  |
| Average | 10,420(54.7%) | 7,257(57.6%) | 3,163(49.0%) |  |
| Relatively high | 507(2.7%) | 365(2.9%) | 142(2.2%) |  |
| Very high | 36(0.2%) | 26(0.2%) | 10(0.2%) |  |
| **Night sleep duration** |  |  |  | <.001 |
| ≤6 h | 9,592(50.4%) | 5,918(47.0%) | 3,674(57.0%) |  |
| 6–8 h | 7,929(41.6%) | 5,679(45.1%) | 2,250(34.9%) |  |
| >8 h | 1,526(8.0%) | 1,001(7.9%) | 525(8.1%) |  |
| **Smoking** |  |  |  | <.001 |
| No | 11,586(60.8%) | 7,505(59.6%) | 4,081(63.3%) |  |
| Yes | 7,461(39.2%) | 5,093(40.4%) | 2,368(36.7%) |  |
| **Drinking** |  |  |  | <.001 |
| No | 12,605(66.2%) | 8,050(63.9%) | 4,555(70.6%) |  |
| Yes | 6,442(33.8%) | 4,548(36.1%) | 1,894(29.4%) |  |
| **Depression** |  |  |  | <.001 |
| No | 10,380(54.5%) | 7,633(60.6%) | 2,747(42.6%) |  |
| Yes | 8,667(45.5%) | 4,965(39.4%) | 3,702(57.4%) |  |
| **Hypertension** |  |  |  | <.001 |
| No | 14,493(76.1%) | 9,826(78.0%) | 4,667(72.4%) |  |
| Yes | 4,554(23.9%) | 2,772(22.0%) | 1,782(27.6%) |  |
| **Dyslipidaemia** |  |  |  | <.001 |
| No | 17,241(90.5%) | 11,481(91.1%) | 5,760(89.3%) |  |
| Yes | 1,806(9.5%) | 1,117(8.9%) | 689(10.7%) |  |
| **Diabetes** |  |  |  | <.001 |
| No | 17,934(94.2%) | 11,986(95.1%) | 5,948(92.2%) |  |
| Yes | 1,113(5.8%) | 612(4.9%) | 501(7.8%) |  |
| **Liver disease** |  |  |  | <.001 |
| No | 18,288(96.0%) | 12,167(96.6%) | 6,121(94.9%) |  |
| Yes | 759(4.0%) | 431(3.4%) | 328(5.1%) |  |
| **Heart disease** |  |  |  | <.001 |
| No | 16,770(88.0%) | 11,292(89.6%) | 5,478(84.9%) |  |
| Yes | 2,277(12.0%) | 1,306(10.4%) | 971(15.1%) |  |
| **Stroke** |  |  |  | <.001 |
| No | 18,633(97.8%) | 12,389(98.3%) | 6,244(96.8%) |  |
| Yes | 414(2.2%) | 209(1.7%) | 205(3.2%) |  |
| **Kidney disease** |  |  |  | <.001 |
| No | 17,847(93.7%) | 11,938(94.8%) | 5,909(91.6%) |  |
| Yes | 1,200(6.3%) | 660(5.2%) | 540(8.4%) |  |
| **Stomach or other digestive disease** |  |  |  | <.001 |
| No | 14,815(77.8%) | 10,131(80.4%) | 4,684(72.6%) |  |
| Yes | 4,232(22.2%) | 2,467(19.6%) | 1,765(27.4%) |  |
| **Memory-related disease** |  |  |  | <.001 |
| No | 18,800(98.7%) | 12,494(99.2%) | 6,306(97.8%) |  |
| Yes | 247(1.3%) | 104(0.8%) | 143(2.2%) |  |
| **Arthritis or rheumatism** |  |  |  | <.001 |
| No | 12,844(67.4%) | 9,063(71.9%) | 3,781(58.6%) |  |
| Yes | 6,203(32.6%) | 3,535(28.1%) | 2,668(41.4%) |  |
| **Menopause** |  |  |  | <.001 |
| No | 2,812(14.8%) | 1,966(15.6%) | 846(13.1%) |  |
| Yes | 7,115(37.4%) | 4,166(33.1%) | 2,949(45.7%) |  |
| Not applicable | 9,120(47.9%) | 6,466(51.3%) | 2,654(41.2%) |  |
| **Prostatic diseases** |  |  |  | <.001 |
| No | 8,191(43.0%) | 5,894(46.8%) | 2,297(35.6%) |  |
| Yes | 929(4.9%) | 572(4.5%) | 357(5.5%) |  |
| Not applicable | 9,927(52.1%) | 6,132(48.7%) | 3,795(58.8%) |  |
| **Hearing impairment** |  |  |  | <.001 |
| Poor | 2,537(13.3%) | 996(7.9%) | 1,541(23.9%) |  |
| Fair | 7,816(41.0%) | 4,983(39.6%) | 2,833(43.9%) |  |
| Good | 5,579(29.3%) | 4,141(32.9%) | 1,438(22.3%) |  |
| Very good | 2,769(14.5%) | 2,185(17.3%) | 584(9.1%) |  |
| Excellent | 346(1.8%) | 293(2.3%) | 53(0.8%) |  |
| **Pain** |  |  |  | <.001 |
| No | 13,213(69.4%) | 9,575(76.0%) | 3,638(56.4%) |  |
| Yes | 5,834(30.6%) | 3,023(24.0%) | 2,811(43.6%) |  |
| **Weigh change** |  |  |  | <.001 |
| Don’t know | 968(5.1%) | 518(4.1%) | 450(7.0%) |  |
| No | 15,869(83.3%) | 10,734(85.2%) | 5,135(79.6%) |  |
| Yes, first gained and then lost weight | 117(0.6%) | 83(0.7%) | 34(0.5%) |  |
| Yes, first lost and then gained weight | 114(0.6%) | 79(0.6%) | 35(0.5%) |  |
| Yes, only gained weight | 644(3.4%) | 426(3.4%) | 218(3.4%) |  |
| Yes, only lost weight | 1,335(7.0%) | 758(6.0%) | 577(8.9%) |  |
| **Health status**  **during childhood** |  |  |  | <.001 |
| Poor | 1,321(6.9%) | 680(5.4%) | 641(9.9%) |  |
| Fair | 3,406(17.9%) | 2,179(17.3%) | 1,227(19.0%) |  |
| Good | 5,082(26.7%) | 3,278(26.0%) | 1,804(28.0%) |  |
| Very Good | 7,227(37.9%) | 4,989(39.6%) | 2,238(34.7%) |  |
| Excellent | 2,011(10.6%) | 1,472(11.7%) | 539(8.4%) |  |
| **self-expectations of health status** |  |  |  | <.001 |
| Almost impossible | 1,685(8.8%) | 850(6.7%) | 835(12.9%) |  |
| Not very likely | 3,804(20.0%) | 2,078(16.5%) | 1,726(26.8%) |  |
| Maybe | 6,886(36.2%) | 4,828(38.3%) | 2,058(31.9%) |  |
| Very likely | 3,101(16.3%) | 2,231(17.7%) | 870(13.5%) |  |
| Almost certain | 3,571(18.7%) | 2,611(20.7%) | 960(14.9%) |  |
| **BMI** |  |  |  | <.001 |
| Underweight | 1,247(6.5%) | 760(6.0%) | 487(7.6%) |  |
| Normal weight | 9,856(51.7%) | 6,488(51.5%) | 3,368(52.2%) |  |
| Overweight | 5,823(30.6%) | 3,915(31.1%) | 1,908(29.6%) |  |
| Obese | 2,121(11.1%) | 1,435(11.4%) | 686(10.6%) |  |
| **Hand grip strength** | 29 (23,37) | 31 (24,38) | 27 (21,34) | <.001 |
| **Waist(cm)** | 85 (78,92) | 85 (78,92) | 85 (78,92) | <.001 |
| **White blood cell(10^9/L)** | 5.97 (4.95,7.20) | 6.00 (4.98,7.20) | 5.90 (4.90,7.20) | .4 |
| **Platelets(10^9/L)** | 206 (163,254) | 207 (163,255) | 204 (162,252) | .09 |
| **Glycated hemoglobin (%)** | 5.10 (4.90,5.40) | 5.10 (4.90,5.40) | 5.10 (4.90,5.50) | .05 |
| **Haemoglobin(g/dL)** | 14.30 (13.00,15.60) | 14.30 (13.10,15.60) | 14.10 (12.90,15.30) | <.001 |
| **Glucose(mmol/L)** | 5.67 (5.22,6.28) | 5.66 (5.22,6.26) | 5.69 (5.24,6.32) | .02 |
| **Total cholesterol(mmol/L)** | 4.91 (4.31,5.56) | 4.89 (4.30,5.55) | 4.93 (4.32,5.58) | .02 |
| **Triglycerides(mmol/L)** | 1.20 (0.86,1.78) | 1.21 (0.86,1.80) | 1.20 (0.86,1.76) | .4 |
| **High-density lipoprotein**  **cholesterol(mmol/L)** | 1.27 (1.03,1.54) | 1.26 (1.03,1.53) | 1.28 (1.04,1.55) | .004 |
| **Low-density lipoprotein**  **cholesterol(mmol/L)** | 2.93 (2.39,3.53) | 2.92 (2.39,3.51) | 2.95 (2.40,3.57) | .07 |
| **House structure** |  |  |  | <.001 |
| Modern | 8,878(46.6%) | 6,224(49.4%) | 2,654(41.2%) |  |
| traditional | 9,702(50.9%) | 6,100(48.4%) | 3,602(55.9%) |  |
| Temporary | 17(0.1%) | 11(0.1%) | 6(0.1%) |  |
| Other | 450(2.4%) | 263(2.1%) | 187(2.9%) |  |
| **Heating energy** |  |  |  | <.001 |
| Solar | 432(2.3%) | 327(2.6%) | 105(1.6%) |  |
| Coal | 5,646(29.6%) | 3,761(29.9%) | 1,885(29.2%) |  |
| Natural gas | 418(2.2%) | 296(2.3%) | 122(1.9%) |  |
| Liquefied Petroleum Gas | 388(2.0%) | 277(2.2%) | 111(1.7%) |  |
| Electric | 3,241(17.0%) | 2,338(18.6%) | 903(14.0%) |  |
| Crop residue/Wood burning | 5,424(28.5%) | 3,330(26.4%) | 2,094(32.5%) |  |
| Other | 3,498(18.4%) | 2,269(18.0%) | 1,229(19.1%) |  |
| **Cooking energy** |  |  |  | <.001 |
| Clear Fuel | 9,825(51.6%) | 6,015(47.7%) | 3,810(59.1%) |  |
| Non-clear Fuel | 9,081(47.7%) | 6,486(51.5%) | 2,595(40.2%) |  |
| Other/Not cooking | 141(0.7%) | 97(0.8%) | 44(0.7%) |  |
| **Room temperature** |  |  |  | <.001 |
| Very hot | 331(1.7%) | 195(1.5%) | 136(2.1%) |  |
| Hot | 1,821(9.6%) | 1,134(9.0%) | 687(10.7%) |  |
| Bearable | 16,050(84.3%) | 10,732(85.2%) | 5,318(82.5%) |  |
| Cold | 654(3.4%) | 406(3.2%) | 248(3.8%) |  |
| Very cold | 89(0.5%) | 51(0.4%) | 38(0.6%) |  |
| Not applicable | 102(0.5%) | 80(0.6%) | 22(0.3%) |  |
| ^a^n (%); Median (25%,75%).  ^b^Pearson's Chi-squared test; Wilcoxon rank sum test. | | | |  |

**Table S2.** The prevalence of VI by province in China from CHARLS (2011-2018) four waves.

| Provinces | Total | VI | Prevalence (%) |
| --- | --- | --- | --- |
| Beijing | 101 | 14 | 13.9% |
| Xinjiang | 116 | 23 | 19.8% |
| Guangxi | 658 | 147 | 22.3% |
| Guizhou | 208 | 52 | 25.0% |
| Liaoning | 607 | 155 | 25.5% |
| Zhejiang | 800 | 217 | 27.1% |
| Heilongjiang | 414 | 122 | 29.5% |
| Hunan | 858 | 254 | 29.6% |
| Guangdong | 1021 | 309 | 30.3% |
| Shanghai | 83 | 26 | 31.3% |
| Shaanxi | 611 | 197 | 32.2% |
| Tianjin | 162 | 53 | 32.7% |
| Gansu | 457 | 153 | 33.5% |
| Fujian | 521 | 176 | 33.8% |
| Jiangsu | 909 | 308 | 33.9% |
| Shandong | 1610 | 547 | 34.0% |
| Inner Mongolia | 939 | 321 | 34.2% |
| Hebei | 815 | 279 | 34.2% |
| Yunnan | 1168 | 400 | 34.2% |
| Jilin | 425 | 148 | 34.8% |
| Shanxi | 553 | 199 | 36.0% |
| Jiangxi | 999 | 362 | 36.2% |
| Henan | 1450 | 534 | 36.8% |
| Hubei | 595 | 223 | 37.5% |
| Sichuan | 1619 | 654 | 40.4% |
| Anhui | 930 | 390 | 41.9% |
| Chongqing | 265 | 117 | 44.2% |
| Qinghai | 153 | 69 | 45.1% |

**Table S3.** Analysis of differences in predictive factors between testing and training datasets.

| Characteristic | Test, N = 4,761^a^ | Train, N = 14,286^a^ | *p*-value^b^ |
| --- | --- | --- | --- |
| **Age** | -0.07 (-0.88, 0.63) | -0.07 (-0.88, 0.63) | >.9 |
| **Gender** |  |  | .6 |
| Female | 2,467 (52%) | 7,460 (52%) |  |
| Male | 2,294 (48%) | 6,826 (48%) |  |
| **Standard of living** |  |  | .9 |
| Average | 2,590 (54%) | 7,830 (55%) |  |
| Poor | 565 (12%) | 1,695 (12%) |  |
| Relatively high | 119 (2.5%) | 388 (2.7%) |  |
| Relatively poor | 1,477 (31%) | 4,347 (30%) |  |
| Very high | 10 (0.2%) | 26 (0.2%) |  |
| **Region** |  |  | .8 |
| Central | 1,599 (34%) | 4,716 (33%) |  |
| East | 1,734 (36%) | 5,261 (37%) |  |
| West | 1,428 (30%) | 4,309 (30%) |  |
| **Education level** |  |  | .6 |
| Elementary school | 1,050 (22%) | 3,063 (21%) |  |
| High school or above | 643 (14%) | 1,952 (14%) |  |
| Less than elementary school | 2,055 (43%) | 6,313 (44%) |  |
| Middle school | 1,013 (21%) | 2,958 (21%) |  |
| **Smoking** | 1,873 (39%) | 5,588 (39%) | .8 |
| **Drinking** | 1,666 (35%) | 4,776 (33%) | .049 |
| **Night sleep duration** | -0.20 (-0.74, 0.88) | -0.20 (-0.74, 0.88) | .4 |
| **BMI** |  |  | .7 |
| Normal weight | 2,444 (51%) | 7,412 (52%) |  |
| Obese | 528 (11%) | 1,593 (11%) |  |
| Overweight | 1,460 (31%) | 4,363 (31%) |  |
| Underweight | 329 (6.9%) | 918 (6.4%) |  |
| **Hand grip strength** | -0.06 (-0.73, 0.74) | -0.08 (-0.72, 0.68) | .6 |
| **Waist(cm)** | -0.05 (-0.75, 0.66) | -0.05 (-0.74, 0.68) | .7 |
| **White blood cell(10^9/L)** | -0.14 (-0.62, 0.47) | -0.13 (-0.67, 0.47) | .6 |
| **Platelets(10^9/L)** | -0.07 (-0.67, 0.57) | -0.07 (-0.65, 0.57) | .6 |
| **Glycated hemoglobin (%)** | -0.21 (-0.45, 0.16) | -0.21 (-0.45, 0.16) | .7 |
| **Haemoglobin(g/dL)** | -0.09 (-0.63, 0.55) | -0.04 (-0.63, 0.55) | .8 |
| **Glucose(mmol/L)** | -0.21 (-0.43, 0.09) | -0.21 (-0.43, 0.08) | >.9 |
| **Total cholesterol(mmol/L)** | -0.06 (-0.67, 0.57) | -0.09 (-0.68, 0.57) | .2 |
| **Triglycerides(mmol/L)** | -0.27 (-0.55, 0.21) | -0.27 (-0.57, 0.22) | >.9 |
| **High-density**  **lipoprotein cholesterol(mmol/L)** | -0.11 (-0.72, 0.59) | -0.14 (-0.72, 0.57) | .3 |
| **Low-density**  **lipoprotein cholesterol(mmol/L)** | -0.06 (-0.66, 0.60) | -0.07 (-0.67, 0.60) | .6 |
| **Depression** | 2,173 (46%) | 6,494 (45%) | .8 |
| **Hypertension** | 1,156 (24%) | 3,398 (24%) | .5 |
| **Dyslipidaemia** | 475 (10.0%) | 1,331 (9.3%) | .2 |
| **Diabetes** | 287 (6.0%) | 826 (5.8%) | .5 |
| **Liver disease** | 185 (3.9%) | 574 (4.0%) | .7 |
| **Heart disease** | 546 (11%) | 1,731 (12%) | .2 |
| **Stroke** | 102 (2.1%) | 312 (2.2%) | .9 |
| **Kidney disease** | 314 (6.6%) | 886 (6.2%) | .3 |
| **Stomach or other digestive disease** | 1,056 (22%) | 3,176 (22%) | >.9 |
| **Memory-related disease** | 59 (1.2%) | 188 (1.3%) | .7 |
| **Arthritis or rheumatism** | 1,568 (33%) | 4,635 (32%) | .5 |
| **Menopause** |  |  | .7 |
| No | 685 (14%) | 2,127 (15%) |  |
| Not applicable | 2,294 (48%) | 6,826 (48%) |  |
| Yes | 1,782 (37%) | 5,333 (37%) |  |
| **Prostatic diseases** |  |  | .9 |
| No | 2,060 (43%) | 6,131 (43%) |  |
| Not applicable | 2,467 (52%) | 7,460 (52%) |  |
| Yes | 234 (4.9%) | 695 (4.9%) |  |
| **Hearing impairment** |  |  | .3 |
| Excellent | 85 (1.8%) | 261 (1.8%) |  |
| Fair | 1,952 (41%) | 5,864 (41%) |  |
| Good | 1,349 (28%) | 4,230 (30%) |  |
| Poor | 663 (14%) | 1,874 (13%) |  |
| Very good | 712 (15%) | 2,057 (14%) |  |
| **Pain** | 1,484 (31%) | 4,350 (30%) | .4 |
| **Weigh change** |  |  | .02 |
| Don’t know | 245 (5.1%) | 723 (5.1%) |  |
| No | 3,910 (82%) | 11,959 (84%) |  |
| Yes, first gained and then lost weight | 37 (0.8%) | 80 (0.6%) |  |
| Yes, first lost and then gained weight | 27 (0.6%) | 87 (0.6%) |  |
| Yes, only gained weight | 193 (4.1%) | 451 (3.2%) |  |
| Yes, only lost weight | 349 (7.3%) | 986 (6.9%) |  |
| **Health status during childhood** |  |  | .4 |
| Excellent | 521 (11%) | 1,490 (10%) |  |
| Fair | 834 (18%) | 2,572 (18%) |  |
| Good | 1,308 (27%) | 3,774 (26%) |  |
| Poor | 319 (6.7%) | 1,002 (7.0%) |  |
| Very Good | 1,779 (37%) | 5,448 (38%) |  |
| **self-expectations of health status** |  |  | .3 |
| Almost certain | 898 (19%) | 2,673 (19%) |  |
| Almost impossible | 452 (9.5%) | 1,233 (8.6%) |  |
| Maybe | 1,703 (36%) | 5,183 (36%) |  |
| Not very likely | 917 (19%) | 2,887 (20%) |  |
| Very likely | 791 (17%) | 2,310 (16%) |  |
| **House structure** |  |  | .4 |
| Modern | 2,262 (48%) | 6,616 (46%) |  |
| Other | 104 (2.2%) | 346 (2.4%) |  |
| Temporary | 3 (<0.1%) | 14 (<0.1%) |  |
| traditional | 2,392 (50%) | 7,310 (51%) |  |
| **Heating energy** |  |  | .8 |
| Coal | 1,384 (29%) | 4,262 (30%) |  |
| Crop residue/Wood burning | 1,349 (28%) | 4,075 (29%) |  |
| Electric | 817 (17%) | 2,424 (17%) |  |
| Liquefied Petroleum Gas | 93 (2.0%) | 295 (2.1%) |  |
| Natural gas | 106 (2.2%) | 312 (2.2%) |  |
| Other | 895 (19%) | 2,603 (18%) |  |
| Solar | 117 (2.5%) | 315 (2.2%) |  |
| **Cooking energy** |  |  | .5 |
| Clear Fuel | 2,423 (51%) | 7,402 (52%) |  |
| Non-clear Fuel | 2,305 (48%) | 6,776 (47%) |  |
| Other/Not cooking | 33 (0.7%) | 108 (0.8%) |  |
| **Room temperature** |  |  | .5 |
| Bearable | 4,021 (84%) | 12,029 (84%) |  |
| Cold | 147 (3.1%) | 507 (3.5%) |  |
| Hot | 454 (9.5%) | 1,367 (9.6%) |  |
| Not applicable | 23 (0.5%) | 79 (0.6%) |  |
| Very cold | 25 (0.5%) | 64 (0.4%) |  |
| Very hot | 91 (1.9%) | 240 (1.7%) |  |
| **Vision impairment** | 1,612 (34%) | 4,837 (34%) | >.9 |
| ^a^n (%); Median (IQR). | | | |
| ^b^Pearson's Chi-squared test; Wilcoxon rank sum test; Fisher's exact test. | | | |

**Table S4.** Comparison of machine learning model performance (testing dataset).

| Model | AUC | Accuracy | Precision | TPR | F1 |
| --- | --- | --- | --- | --- | --- |
| GLM | 0.706 | 0.620 | 0.461 | 0.718 | 0.561 |
| GBM | 0.710 | 0.611 | 0.455 | 0.754 | 0.567 |
| DRF | 0.698 | 0.622 | 0.462 | 0.710 | 0.560 |
| DL | 0.698 | 0.589 | 0.439 | 0.782 | 0.563 |
| StackedEnsemble | 0.715 | 0.625 | 0.466 | 0.735 | 0.570 |

AUC, area under the curve; TPR, true positive rate; GLM, generalized linear model; GBM, gradient boosting machine; DRF, distributed random forest; DL, deep learning; StackedEnsemble, GBM-XGBoost-GLM-DL-DRF.

**Table S5.** The hyperparameters used in model training.

| Model | Parameters | Values |
| --- | --- | --- |
| GLM | alpha | 0 |
|  | lambda | 11.036.85 4.262.64 1.64 1.02... |
|  | max iterations | 30 |
| GBM | max_depth | 8 |
|  | ntrees | 36 |
|  | learn_rate | 0.1 |
| DRF | ntrees | 50 |
|  | max_depth | 20 |
| DL | activation | 'RectifierWithDropout' |
|  | hidden | 20 20 20 |
|  | input_dropout_ratio | 0.1 |
|  | l1 | 0 |
|  | l2 | 0 |

GLM, generalized linear model; GBM, gradient boosting machine; DRF, distributed

random forest; DL, deep learning.


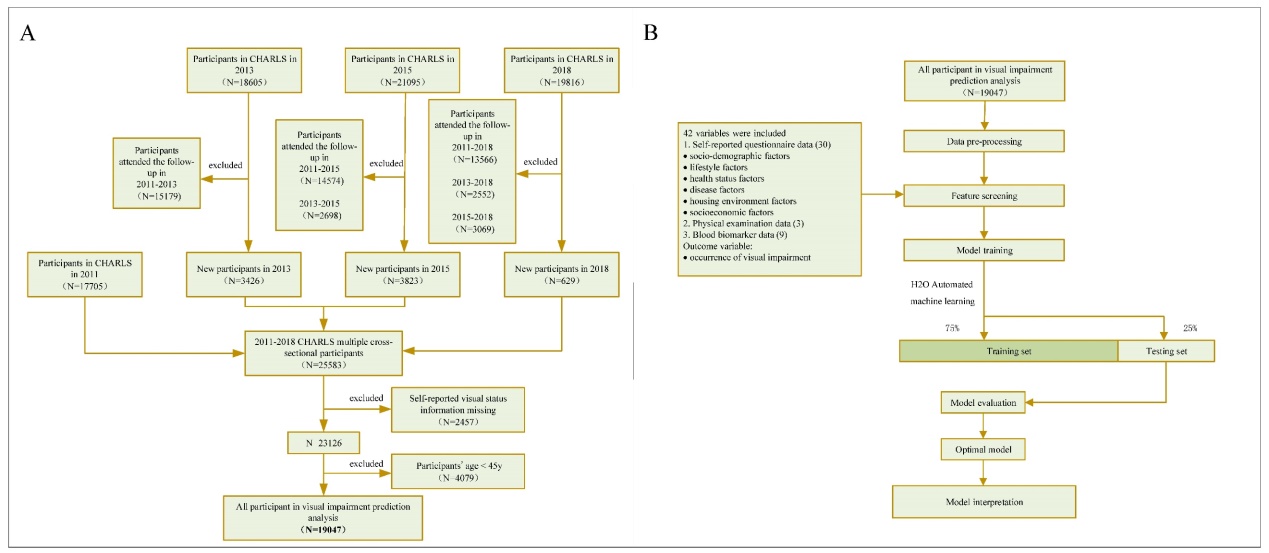


**Figure S1.** Flowchart of this study. A, the sampling process; B, the machine learning process.


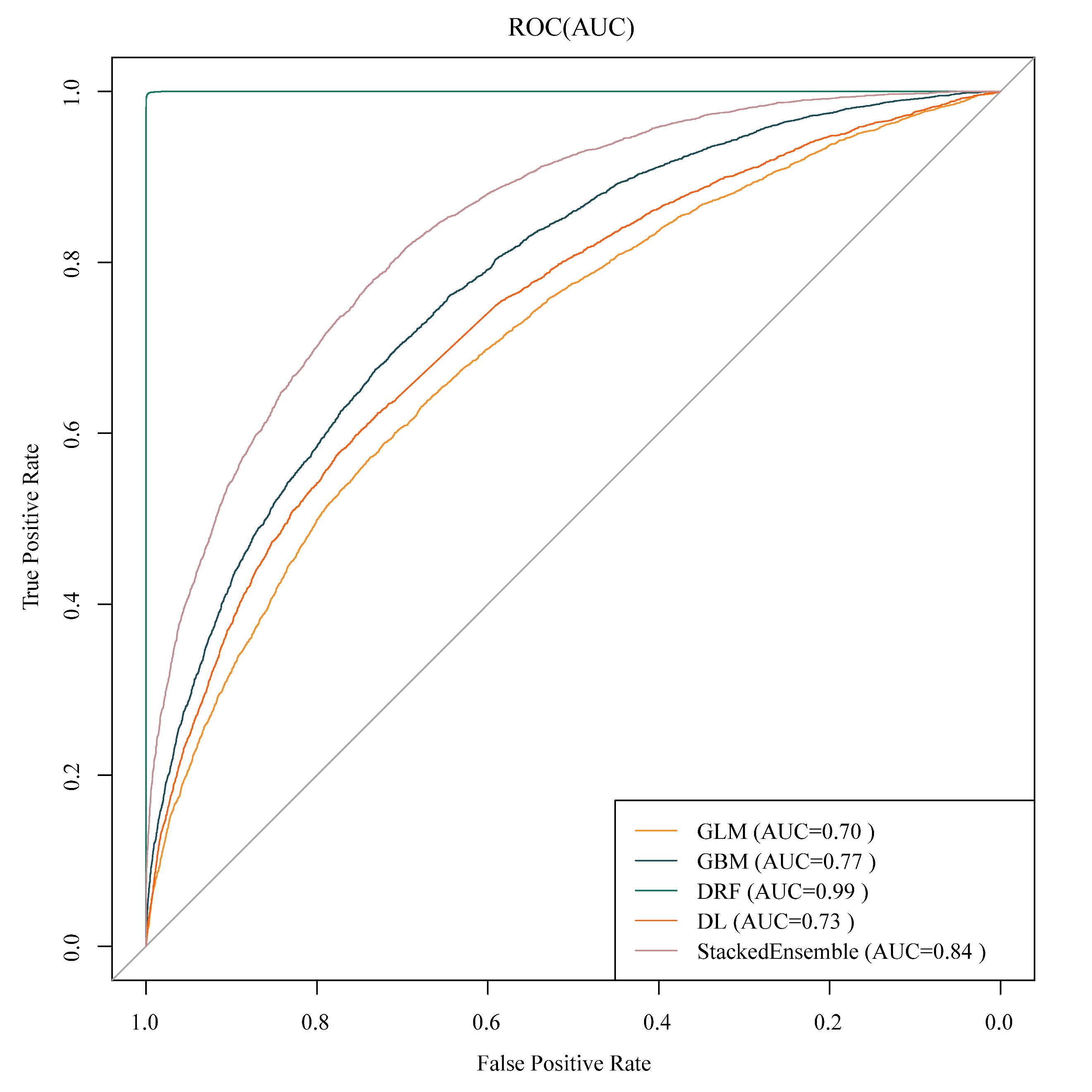


**Figure S2.** Receiver operating characteristic curves of all VI prediction models on the training dataset. AUC, area under the curve; GLM, generalized linear model; GBM, gradient boosting machine; DRF, distributed random forest; DL, deep learning; StackedEnsemble, GBM-XGBoost-GLM-DL-DRF.
